# The Metagenome of an Anaerobic Microbial Community Decomposing Poplar Wood Chips

**DOI:** 10.1371/journal.pone.0036740

**Published:** 2012-05-21

**Authors:** Daniel van der Lelie, Safiyh Taghavi, Sean M. McCorkle, Luen-Luen Li, Stephanie A. Malfatti, Denise Monteleone, Bryon S. Donohoe, Shi-You Ding, William S. Adney, Michael E. Himmel, Susannah G. Tringe

**Affiliations:** 1 Biology Department, Brookhaven National Laboratory, Upton, New York, United States of America; 2 Oak Ridge National Laboratory, BioEnergy Science Center, Oak Ridge, Tennessee, United States of America; 3 Center for Agricultural and Environmental Biotechnology, RTI International, Research Triangle Park, North Carolina, United States of America; 4 DOE Joint Genome Institute, Walnut Creek, California, United States of America; 5 National Renewable Energy Laboratory, Golden, Colorado, United States of America; J. Craig Venter Institute, United States of America

## Abstract

This study describes the composition and metabolic potential of a lignocellulosic biomass degrading community that decays poplar wood chips under anaerobic conditions. We examined the community that developed on poplar biomass in a non-aerated bioreactor over the course of a year, with no microbial inoculation other than the naturally occurring organisms on the woody material. The composition of this community contrasts in important ways with biomass-degrading communities associated with higher organisms, which have evolved over millions of years into a symbiotic relationship. Both mammalian and insect hosts provide partial size reduction, chemical treatments (low or high pH environments), and complex enzymatic ‘secretomes’ that improve microbial access to cell wall polymers. We hypothesized that in order to efficiently degrade coarse untreated biomass, a spontaneously assembled free-living community must both employ alternative strategies, such as enzymatic lignin depolymerization, for accessing hemicellulose and cellulose and have a much broader metabolic potential than host-associated communities. This would suggest that such a community would make a valuable resource for finding new catalytic functions involved in biomass decomposition and gaining new insight into the poorly understood process of anaerobic lignin depolymerization. Therefore, in addition to determining the major players in this community, our work specifically aimed at identifying functions potentially involved in the depolymerization of cellulose, hemicelluloses, and lignin, and to assign specific roles to the prevalent community members in the collaborative process of biomass decomposition. A bacterium similar to *Magnetospirillum* was identified among the dominant community members, which could play a key role in the anaerobic breakdown of aromatic compounds. We suggest that these compounds are released from the lignin fraction in poplar hardwood during the decay process, which would point to lignin-modification or depolymerization under anaerobic conditions.

## Introduction

In recent years, biofuels have attracted great interest as an alternative, renewable source of energy in the face of the ongoing depletion of fossil fuels, our energy dependence on them, and our growing environmental awareness of the negative consequences of burning such fuels. Plant biomass represents a globally abundant carbon source with the potential to provide a sustainable source of mixed sugars for biofuels production [Billion Ton Report; http://feedstockreview.ornl.gov/pdf/billion_ton_vision.pdf]. However, breakthrough technologies are still needed to overcome the critical barriers to developing cost-effective processes for converting recalcitrant biomass to fuels and chemicals, such as the high cost of commercially available lignocellulolytic enzymes and hardware for thermal chemical pretreatment [1].

In nature, cellulosic biomass is decomposed by complex and efficient processes, usually by microbial communities that produce cellulolytic enzymes that function synergistically to decompose plant biomass [2]–[4]. The enzymes primarily involved in plant biomass breakdown are the glycoside hydrolases (also known as glycosidases), which catalyze the hydrolysis of the β (1–4) main chain bonds in cellulose and hemicelluloses; as well as the considerable diversity of side chain bonds in hard wood hemicellulose [5]. In the decomposition of terrestrial biomass, bacterial and fungal communities often occupy various temporal stages of the conversion process, depending on the microenvironment of the decay site [6]–[9].

Prokaryotic microorganisms have evolved and accumulated remarkable physiological and functional diversity, and thereby harbor a major reserve of genetic potential. The traditional method to tap this reservoir of functional potential is by cultivating microorganisms and screening individuals for the requisite phenotypes. However, 95 to 99.9% of microorganisms are not readily cultured by standard laboratory techniques [10]. Metagenomic approaches provide a powerful tool to bypass the limitation of cultivation-based methodologies, as they allow to predict the biochemical potential of a particular community or to prospect novel biocatalysts from environmental samples for experimental validation and characterization [11]–[17], including those involved in biomass decomposition [13], [18]. In the current study, we examined a free-living microbial consortium capable of degrading poplar biomass in the absence of oxygen or other nutrient sources, which we hypothesized, would possess a rich supply of enzymes for degrading cellulose, hemicelluloses and possibly lignin. We generated several hundred megabases of metagenomic sequence data which proved sufficient to assemble draft genomes of dominant community members and annotate several thousand putative biomass degrading genes.

## Results

### Imaging of biomass decomposition

**Figure 1:**
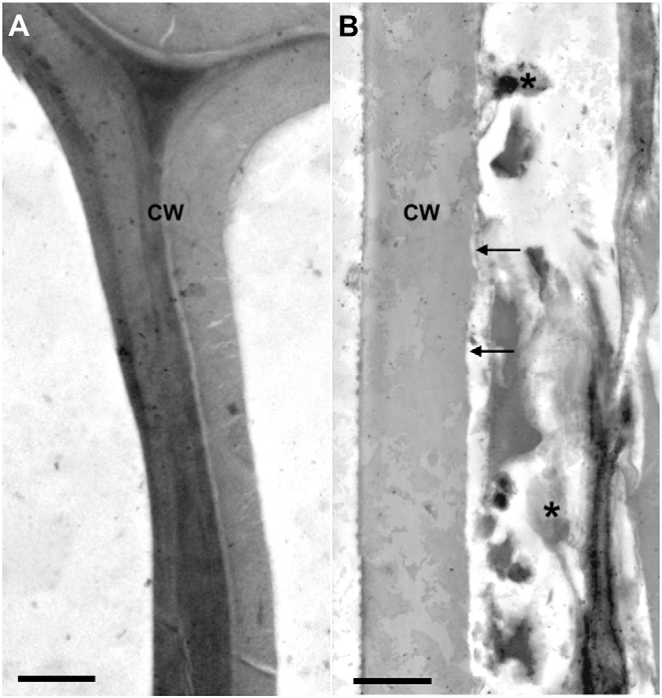
TEM micrographs of control (A) and composted (B) poplar cell walls. The composted cell walls display eroded surfaces (arrows) and accumulation of debris (*). Scale bars = 1 µm.

**Figure 2:**
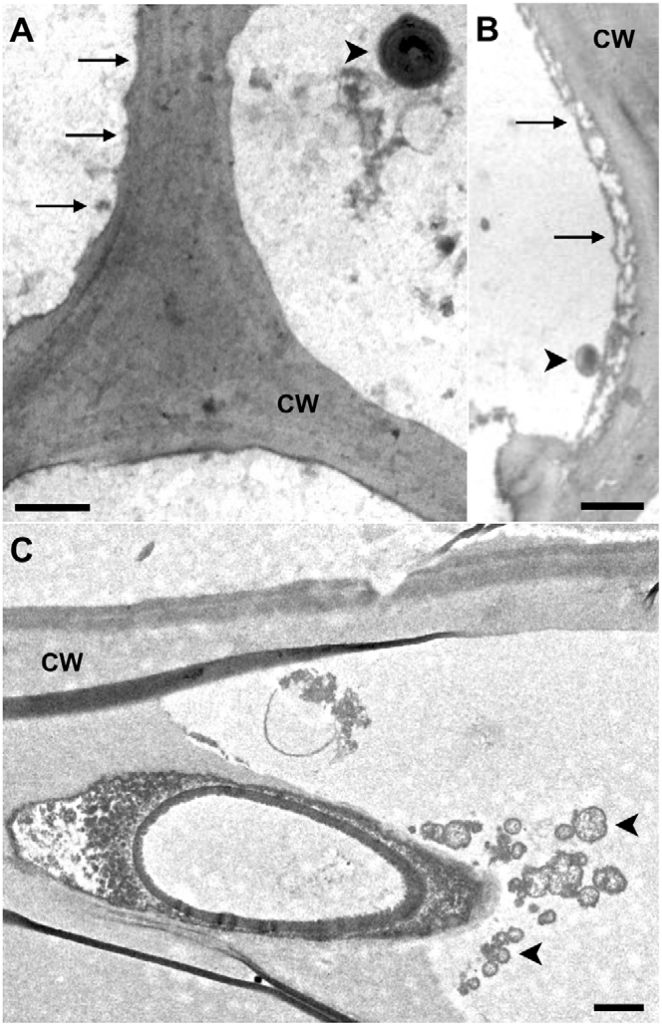
TEM micrographs of composted poplar cell walls. These cell walls display evidence of microbial decay including a scalloped surface (A, B, arrows) and direct microbe attachment to eroded cell wall surfaces (B, C, arrowheads). Scale bars = 500 nm. The arrowheads in Figure 2C point to cellulosomes.

After one year incubation at 30°C in a closed system, which created a gradient from micro-aerobic to anaerobic, the poplar biomass showed strong visual signs of decomposition. The volume of the biomass had compacted approximately 25%, the biomass particles became softer, and the color had changed from light to dark brown (see Fig. S1 and Methods S1 for sample preparation in the supplementary materials). To confirm the decay process Transmission electron microscopy (TEM) was used to examine the structural integrity of the biomass at the cell wall level. By comparing samples from untreated (Fig. 1A) and incubated biomass (Fig. 1B), clear signs of cell wall decomposition were observed. More detailed imaging revealed attached microbial cells (Fig. 2A), indentations in the cell wall (Fig. 2B), and invading cells covered with cellulosome-like structures (Fig. 2C).

**Figure 3:**
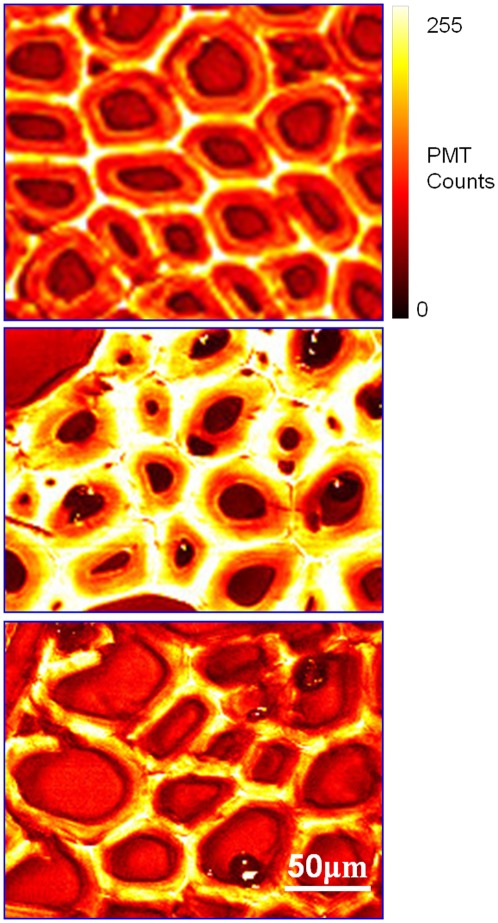
Coherent Raman scattering (CRS) micrographs of poplar cell walls. The lignin signal (1600 cm^−1^) was obtained using coherent anti-Stokes Raman (CARS) microscopy. In the untreated cell walls (a), lignin is unevenly distributed across cell walls, compound middle lamella have most lignin content; when treated in anaerobic condition (b), polysaccharides are consumed by anaerobic microbes, relative lignin signal is increased; and when treated in aerobic condition (c), aerobic microbes consume polysaccharides (cell walls become thinner), and degrade/modify lignin simultaneously, lignin signal retain unchanged compared with untreated cell walls.

To further investigate the effects of bioreactor incubation on cell wall structure, a combination of imaging techniques was used. Coherent anti-Stokes Raman (CARS) microscopy, used to visualize the lignin, showed that microbial digestion under anaerobic conditions resulted in a relative enhancement of the lignin signal (Fig. 3), thus pointing towards preferential degradation of hemicellulose and cellulose. The unchanged thickness of the plant cell walls in the anaerobic samples suggests that lignin breakdown in the bioreactor is limited, but sufficient to gain access to the cellulose and hemicellulose. Degradation of cellulose and hemicellulose was confirmed by immune staining with fluorescent antibodies followed by epifluorescent light microscopy (Fig. S2 and Methods S1). In comparison with untreated biomass, samples from the composted poplar biomass showed a decrease in the fluorescent signals for both cellulose and hemicellulose. CARS microscopy also showed that under aerobic conditions, in the upper part of the bioreactor, polysaccharides and lignin were simultaneously degraded and/or modified, resulting in a thinner plant cell wall that showed relatively unchanged lignin signal intensity as compared to that of untreated cell walls.

### Metagenome sequencing

DNA extracted from the bioreactor material was subjected to extensive 16 S/18 S profiling by V6–V8 pyrotag sequencing (∼1.5 million tags). To characterize the functional diversity and potential of the community, we performed metagenome sequencing on both the Sanger and 454 sequencing platforms (see Methods). A total of approximately 675 Mb of sequence was generated, and assembly resulted in 44,600 contigs and 1.24 M singletons, totaling 382 Mb, that contain a total of 893,380 putative genes. A summary of the metagenome sequencing statistics is provided in Table 1.

**Table 1:** Summary of the metagenome sequencing statistics.

Characteristic	Amount
Number of Raw Reads generated	2,256,739
Raw bases generated	674,959,683 bp
**Assembly Statistics**	
Number of assembled contigs	44,603
Largest contig Size	85,350 bp
Total # large contigs (>500 bp)	34,156
Total # bases large contigs	46,599,402 bp
Average large contig Size	1364 bp
N50 large contig size	2,011
Total assembled contig length	51,160,060 bp
Number of reads completely or partially assembled	934,264
Number of singletons	1,242,991
Total # unassembled reads	1,322,475
Total singleton length	331,198,036 bp
Number of contigs and singletons	1,287,594
Total contig and singleton length	382,358,096 bp
Total # scaffolds	1,105
Total bases in scaffolds	34,278,314 bp
Average scaffold size	31,021 bp
N50 scaffold size	707,250 bp
Largest scaffold size	5,104,732

### Microbial community composition

**Figure 4:**
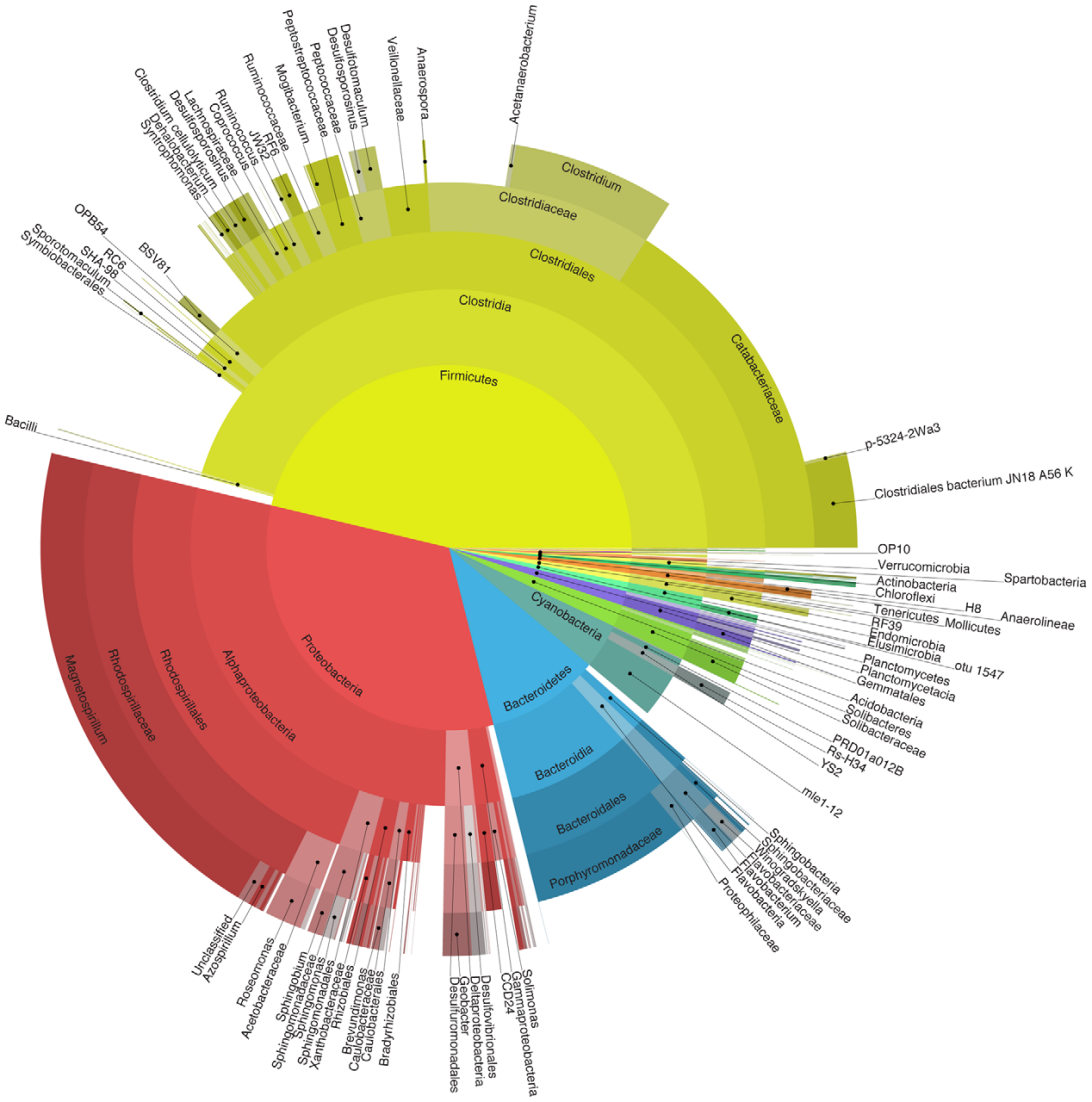
Taxonomic breakdown of the microbiome of an anaerobic microbial community decomposing poplar wood chips based on classified 16 S rRNA gene sequences. Central pie shows percentages by phyla; each outer annulus progressively breaks these down by finer taxonomic levels: class, order, family and genus in the outermost annulus. The pyrotag data were submitted to the Short Read Archive under SRA number SRA045915.

Analysis of 1,516,612 V6–V8 pyrotags (after filtering for length and quality; see methods) indicated the presence of a diverse community degrading the poplar biomass under anaerobic conditions (see Fig. 4). As we found in the metagenome binning, the *Firmicutes* and *Proteobacteria* represented the most abundant microbial phyla (with 45.9% and 32.3%, respectively), followed by the *Bacteroidetes* (9.9%). The *Firmicutes* were dominated by the class *Clostridia* that form the most abundant class of microbes in this community. They are mainly represented by members of the orders *Clostridiales* (37.5%), which was dominated by members of the family of the *Clostridiaceae* (9.9%) and *Catabacteriaceae* (15.8%). Of the *Clostridiales*, species of the genus *Clostridium*, which encompasses numerous highly active anaerobic cell wall degraders (e.g. *C. cellulolyticum* and *C. thermocellum*), comprised nearly 64% of this order (6.3% of the total community).

The *Proteobacteria*, representing 32.4% of the total community, were dominated by the alpha-*Proteobacteria*, which comprise 28.2% of the tag data. Of this class, 22.9% belonged to the order *Rhodospirillales*, the family *Rhodospirillaceae* (21.1%) and the genus *Magnetospirillum* (19.9%), making this the most abundant genus in the community. The *Bacteroidetes* are the third most abundant group of microorganisms, representing 9.9% of the total community. Finally, we also observed a group of Cyanobacteria, which represented approximately 4% of the community. The Archaea formed only a small part of the total pyrotag data, largely because of a mismatch in the forward primer with most archaeal lineages. We found 11,155 tags representing *Euryarchaeota*, of which 10,258 tags could be assigned to the *Methanomicrobia*. Only 678 tags were derived from Eukaryota, indicating that fungi don't play a major role in the decomposition of biomass in this community.

### Metagenome assembly and binning

This deep sequencing effort resulted in significant assembly, such that >52% of the quality filtered, non-redundant reads assembled into 34,156 contigs greater than 500 bp in length totaling 46.6 Mb in size. While many of these contigs were small (average size 1364 bp), there were many well-assembled contigs of up to ∼80 kb and ∼20× depth when all the data were assembled with Newbler [19]. Moreover, there were several scaffolds over 1 Mb in size that could represent draft genomes of bioreactor populations.

Phylogenetic marker genes, including 16 S ribosomal RNA and ribosomal protein genes, were identified in the sequence data and the contigs/scaffolds in which they resided and used to train a phylogenetic classifier using ClaMS (Classifier for Metagenome Sequences; http://clams.jgi-psf.org/). Binning of the larger contigs (at least 1 kb) using trinucleotide distributions resulted in the formation of six well defined clusters thought to represent relatives of *Magnetospirillum*, *Bacteroides*, two distinct *Clostridiales*, *Cyanobacteria* and a cluster representing Archaea of the class *Methanomicrobia* (Table 2). For 36% of the contigs greater than 1 kb in length, no match to any of the major clusters was obtained. Dominance of the biomass degrading community by species belonging to the classes *Clostridia*, *Bacteroidetes* and *Methanomicrobiales* was also described for the metagenome of a microbial community of a production-scale biogas plant fermenter [20]. In the case of the poplar biomass degrading community the dominance of *Magnetospirillum* (present at ∼10× to 20× coverage in the metagenome assembly) putatively involved in biomass decomposition is unique, as is the absence of fungi. This point opens the possibility for the existence of new mechanisms for biomass decomposition, including lignin depolymerization, under anaerobic conditions. These data largely confirmed the results of community profiling by pyrotag sequencing, with the exception of *Methanomicrobiales*, an archaeal group which was poorly targeted by the pyrotag primers used.

**Table 2:** Microbial representation from the decomposing poplar wood chips.

Bin	Contigs	Size (Mb)	G+C %	Depth
*Magnetospirillum*	713	3.91	64.9	10.8×
*Bacteroides*	1125	6.7	45.9	6.6×
*Clostridiales* 1	682	2.67	49.9	5.8×
*Cyanobacteria*	1054	5.1	50.6	5.7×
*Clostridiales* 2	1016	2.97	55.3	5.4×
*Methanomicrobia*	1395	4.04	54.1	5.3×

### Bioprospecting for GHases and other enzymes involved in biomass breakdown

**Figure 5:**
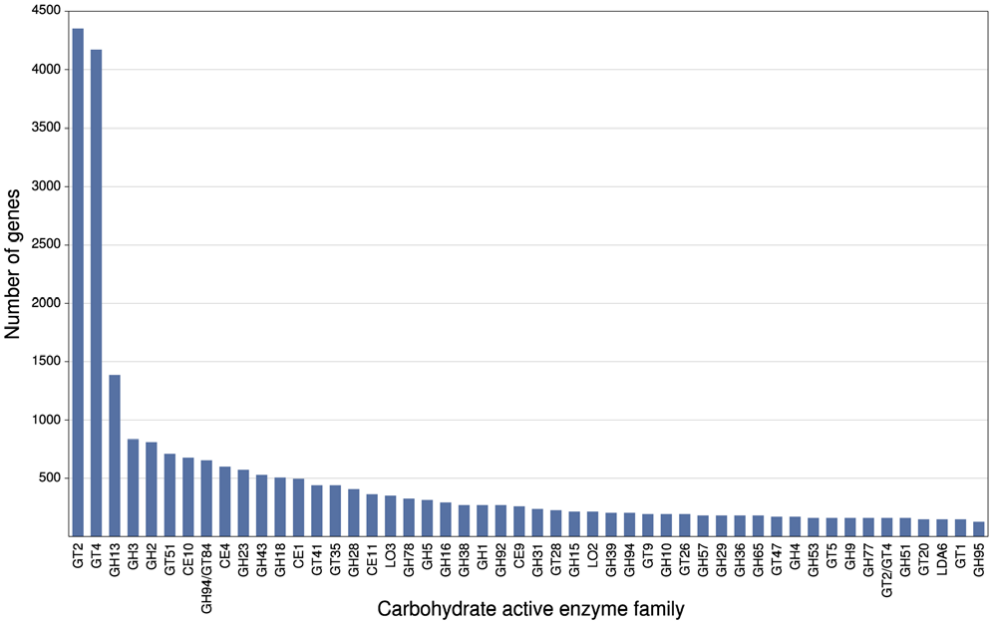
Number of occurrences for the 23 most dominant carbohydrate active enzyme families. In total, these families contained 19114 candidate genes, representing 67.6% of the putative carbohydrate active enzyme family genes.

Genes whose enzymes were putatively involved in the decomposition of recalcitrant plant biomass or modifying carbohydrates were identified based on their similarity to carbohydrate active enzymes (glycoside hydrolases, glycosyl transferases) and fungal oxidoreductases as described in the CAZy (http://www.cazy.org/) [22] and FOLy (http://foly.esil.univ-mrs.fr) [21] databases, respectively. Of the 888,455 protein-coding genes identified by IMG/M, 28,793 candidate genes were identified based on BlastP homology with CAZy or FOLy genes (e-value<1e-10), representing a total of 230 gene families. The 22 most dominant gene families contained 19,510 candidate genes, representing 67.8% of the genes. Candidate genes belonging to the glycosyl transferase families GT2 (4,354) and GT4 (4,178) were the most abundant, followed by members of the glycoside hydrolase families GH13 (1,381), GH3 (832) and GH2 (814) (see Fig. 5). The glycosyl transferases are ubiquitous enzymes that catalyze the attachment of sugars to a glycone [23], [24] and are not thought to be involved in cellulose and hemicellulose hydrolysis. The dominance of candidate members of the glycoside hydrolase family 13 is not surprising, as is the largest sequence-based family of glycoside hydrolases and groups together a number of different enzyme activities and substrate specificities acting on α-glycosidic bonds, including hydrolases, transglycosidases and isomerases [25]. The Family 3 enzymes, also quite abundant in this community, have been classified as β-D-glucosidases, α-L-arabinofuranosidases, β-D-xylopyranosidases and *N*-acetyl-β-D-glucosaminidases [26]. In many cases, the enzymes have dual or broad substrate specificities with respect to monosaccharide residues, linkage position and chain length of the substrate, such as both α-L-arabinofuranosidase and β-D-xylopyranosidase activity [27]. The most common activities for bacterial Family 2 glycoside hydrolases include β-D-galactosidases, β-glucuronidases, β-D-mannosidases, and exo-β-glucosaminidases. Members of these GHase families seem to play a key role in the breakdown of hemicellulose and disaccharides. Members of cellulose degrading gene families GH5, 9, 44, 46, 51, and 74 (which also degrades xyloglucan) are represented in the consortium, although not at high levels. The GH family 10 enzyme, known to hydrolyze the β-(1–4) glycosidic linkages in xylan, is also observed. Recently, functional verification of a small subset of the predicted biomass-degrading genes, which were selected based on homology to plant biomass-degrading GHase families/activities of interest and the quality of their sequences, was performed, and resulted in the cloning of four novel GHases that could be successfully expressed in *E. coli* [28].

Many candidate genes displayed some degree of homology to fungal oxidase families catalogued in the FOLy database including the Families LO3 (including cellobiose dehydrogenases and dihydrolipoamide dehydrogenases; 335 genes), LO2 (peroxidases, 231 genes) and LO1 (multi-copper oxidases, 35 genes) [22]. However, sequence composition based binning, as well as closest blast hits, indicated that most, if not all, of these genes were derived from bacterial members of the community. Indeed, very few putative fungal genes involved in biomass decomposition were found, which is consistent with the apparent lack of candidate fungal GHase Family 7 members in this community or fungal 18 S rRNA genes as determined by pyrotag sequencing.

### Comparison to other metagenomes

To put the biomass degrading potential of the bioreactor community decaying poplar woodchips in perspective, we compared it to metagenome data from several other communities expected to have some biomass degrading activity, including the microbiomes of compost [29], rhizosphere soils from maize, miscanthus and switchgrass (unpublished, see GOLD CARD Gm00349), the top and bottom part of the fungal garden of the leaf-cutter ant (*Atta colombica*) [30], cow rumen [18], and the gut communities from termite [31], wallaby [32], canine [33], human [34] and mouse [35].

**Figure 6:**
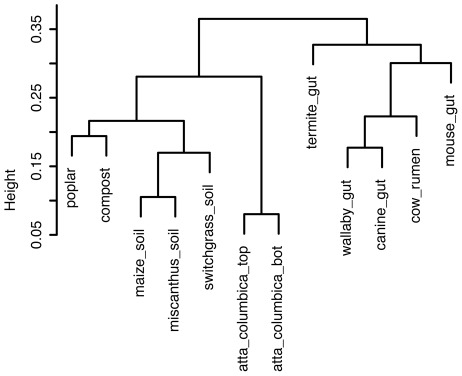
Calculated correlation distances between the metagenomes of the microbiomes associated with the poplar biomass decay community, compost, rhizosphere soils from maize, miscanthus and switchgrass, the top and bottom part of the fungal garden of the leaf-cutter ant (*Atta colombica*), cow rumen, and the gut communities from termite, wallaby, canine, human and mouse. The distribution and relative abundance of glycoside hydrolase families was used as variables for metagenome comparison on the level of functionality related to biomass breakdown. This information was obtained for the various metagenome sequence sets by performing Blastx against the CAZy database, and was subsequently used to calculate the correlation distances between the various metagenomes.

Rather than comparing various metagenomes on the species level, we used the distribution and relative abundance of glycoside hydrolase families as variables for metagenome comparison on the level of functionality related to biomass breakdown. This information was obtained for the various metagenome sequence sets by performing Blastx searches against the CAZy database (to avoid biases from different gene prediction algorithms used), and was subsequently used to calculate the correlation distances between the various metagenomes (Figure 6). The use of datasets that differ significantly in the amount of sequence data was accounted for by (i) performing CAZy blasts with overlapping 300 bp subsequences from the assembled contigs and unassembled reads, to avoid a bias toward gene discovery in longer sequences; (ii) multiplying by read depth when hits occurred on assembled contigs to account for abundance in raw data; (iii) normalizing all counts to the total amount of sequence data weighted by contig depth (a sum of total bases multiplied by depth for each contig). Our analysis indicated that total frequency of CAZy database hits per million nucleotides was relatively consistent across datasets, suggesting that total dataset size was not having a dramatic effect. Moreover, we did not see a tendency of similar size datasets to cluster together.

Clearly, the gut communities form a distinct group from the free-living biomass decay communities, even though genes involved in host-symbiont interactions were not part of the analysis. Notably, this is the case despite the very different sequencing technologies and read lengths used for these projects (e.g. Sanger for termite and wallaby; 454 for human, dog and mouse; and Illumina for cow rumen). In addition, the microbial communities found in compost and decaying poplar woodchips show, at least on the level of glycoside hydrolase abundance, a close affiliation. A close correlation is also observed for the glycoside hydrolase distributions among the maize, miscanthus and switchgrass associated rhizosphere communities, as well as the microbiomes of the top and bottom part of the fungal garden of the leaf-cutter ant (*Atta colombica*) [30]. Within the gut communities, the distribution of GHases in the termite hindgut microbial community is distinct from that seen in mammals.

**Figure 7:**
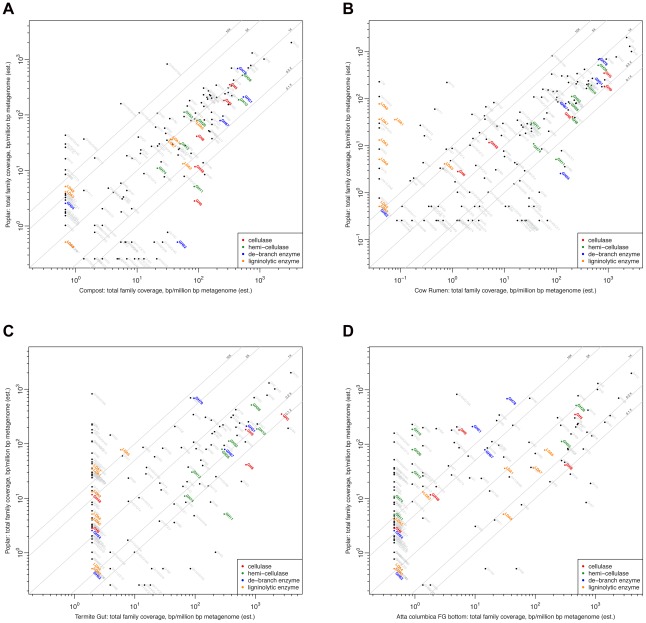
Comparison for the various metagenomes of the total coverage per family of biomass modifying enzymes. This included cellulases, hemicellulases, debranching enzymes and enzymes homologous to lignolytic enzymes. The comparisons for the poplar woodchip bioreactor community with compost, cow rumen, termite hindgut, and fungal garden bottom are presented in Figures 7A, 7B, 7C and 7D, respectively.

To reveal the basis for these differences on a gene level, the total coverage per family of biomass modifying enzymes, which included cellulases, hemicellulases, debranching enzymes [21] and enzymes homologous to lignolytic enzymes [22], was compared for the various metagenomes. The comparisons for the poplar woodchip bioreactor community with those in compost, cow rumen, termite hindgut, and fungal garden bottom are presented in Figures 7A, 7B, 7C and 7D, respectively. Strikingly, a near-complete lack of putative lignin degrading enzymes was shared by all the gut communities examined (wallaby, dog, human, mouse and even cow rumen).

### GHase distribution over phylogenetic groups

Cultivation-based approaches did not result in the isolation of members of the six major phylogenetic groups as identified based on binning of the major metagenome contigs. Although we successfully isolated various members of the genus *Clostridium*, sequencing of their 16 S rRNA genes placed them outside the two dominant *Clostridiales* clusters. In order to determine the putative roles of members of the six dominant microbial clusters in poplar biomass decomposition, the distribution of the biomass modifying enzyme families, which included cellulases, hemicellulases, debranching enzymes and enzymes homologous to lignolytic enzymes, was compared for the various clusters. The data are presented in Table 3.

**Table 3:** Distribution of glycoside hydrolase and lignin peroxidase families over the phylogenetic groups of binned contigs.

Family	*Cyanobacteria*	*Methanomicrobia*	*Bacteroides*	*Magnetospirillum*	*Clostridiales*	*Clostridiales* 2	No match
GH1	419.23	504.01	23.67	593.99	0.00	467.39	180.62
GH1/GH95	0.00	0.00	47.34	0.00	55.20	51.93	72.25
GH2	0.00	588.01	5018.17	0.00	3201.55	0.00	830.85
GH3	493.21	0.00	2106.69	66.00	2925.56	2077.3	72.25
GH4	0.00	0.00	0.00	0.00	883.19	0.00	108.37
GH5	246.61	84.00	449.74	33.00	386.39	363.53	577.99
GH5/CE2	0.00	0.00	0.00	0.00	55.20	0.00	0.00
GH9	0.00	378.01	307.72	0.00	0.00	467.39	0.00
GH10/CE1	0.00	0.00	23.67	0.00	0.00	0.00	0.00
GH10	0.00	84.00	378.73	0.00	0.00	519.33	72.25
GH12	0.00	0.00	47.34	99.00	0.00	0.00	0.00
GH13	1307.02	1680.02	2035.67	3860.93	1987.17	103.87	1264.34
GH13/GH77	0.00	336.00	0.00	99.00	0.00	0.00	0.00
GH13/GH14	0.00	42.00	0.00	0.00	0.00	0.00	0.00
GH14	73.98	126.00	0.00	0.00	0.00	0.00	0.00
GH15	0.00	0.00	307.72	296.99	0.00	0.00	288.99
GH16	0.00	84.00	307.72	560.99	0.00	0.00	252.87
GH16/GH43	0.00	0.00	236.71	0.00	0.00	0.00	0.00
GH17	0.00	0.00	0.00	296.99	0.00	0.00	0.00
GH17/GT2	0.00	0.00	0.00	593.99	0.00	0.00	0.00
GH18	49.32	420.01	284.05	165.00	110.40	830.92	288.99
GH19	0.00	0.00	0.00	0.00	0.00	51.93	0.00
GH20	0.00	0.00	1041.51	0.00	827.99	0.00	0.00
GH23	197.29	0.00	686.45	1616.97	0.00	519.33	252.87
GH24	0.00	0.00	23.67	33.00	0.00	0.00	0.00
GH25	98.64	0.00	94.68	0.00	0.00	51.93	0.00
GH26	0.00	0.00	473.41	0.00	0.00	0.00	0.00
GH28	493.21	0.00	1041.51	296.99	607.19	363.53	469.61
GH29	0.00	0.00	1088.85	0.00	386.39	0.00	0.00
GH30	0.00	378.01	236.71	33.00	0.00	0.00	0.00
GH31	468.55	0.00	402.40	0.00	0.00	155.80	180.62
GH32	0.00	0.00	260.38	0.00	276.00	0.00	0.00
GH33	0.00	0.00	710.12	0.00	276.00	0.00	0.00
GH33/GH78	0.00	0.00	165.69	0.00	0.00	0.00	0.00
GH35	0.00	0.00	686.45	0.00	0.00	0.00	0.00
GH36	98.64	126.00	307.72	0.00	110.40	0.00	180.62
GH38	98.64	0.00	449.74	0.00	1711.17	0.00	72.25
GH39	0.00	0.00	94.68	0.00	0.00	0.00	0.00
GH42/GH43	0.00	0.00	307.72	0.00	0.00	0.00	0.00
GH42	0.00	0.00	355.06	0.00	0.00	0.00	0.00
GH43/GH95	0.00	0.00	142.02	0.00	0.00	0.00	0.00
GH43	147.96	84.00	3455.91	0.00	331.19	0.00	180.62
GH44	0.00	0.00	23.67	0.00	0.00	0.00	0.00
GH48	0.00	0.00	0.00	0.00	0.00	0.00	36.12
GH50	0.00	0.00	47.34	0.00	0.00	0.00	0.00
GH51	0.00	0.00	828.47	0.00	0.00	0.00	0.00
GH53	0.00	0.00	284.05	165.00	0.00	51.93	72.25
GH55	0.00	0.00	23.67	0.00	0.00	0.00	0.00
GH57/GT4	49.32	0.00	0.00	0.00	0.00	0.00	0.00
GH57	271.27	126.00	189.37	0.00	0.00	0.00	144.50
GH65	0.00	42.00	0.00	0.00	827.99	51.93	0.00
GH67	0.00	0.00	284.05	0.00	0.00	0.00	0.00
GH71	0.00	0.00	23.67	0.00	0.00	0.00	0.00
GH73	24.66	0.00	165.69	0.00	0.00	155.80	216.74
GH74	0.00	0.00	0.00	0.00	0.00	0.00	108.37
GH75	24.66	0.00	0.00	0.00	0.00	0.00	0.00
GH76	49.32	0.00	165.69	0.00	0.00	0.00	0.00
GH77	197.29	420.01	378.73	494.99	441.59	0.00	36.12
GH78	0.00	0.00	3810.97	0.00	1269.58	0.00	0.00
GH84	0.00	0.00	0.00	0.00	0.00	0.00	36.12
GH86	0.00	0.00	0.00	0.00	0.00	0.00	72.25
GH87	0.00	0.00	0.00	0.00	110.40	0.00	0.00
GH88	0.00	0.00	165.69	0.00	0.00	0.00	0.00
GH92	0.00	42.00	5065.52	0.00	220.80	519.33	72.25
GH93	123.30	0.00	118.35	0.00	0.00	0.00	180.62
GH94/GT84	838.46	336.00	473.41	725.99	331.19	2285.04	650.23
GH94	73.98	0.00	0.00	0.00	0.00	830.92	36.12
GH95	0.00	0.00	1136.19	0.00	0.00	0.00	0.00
GH97	0.00	0.00	1254.54	0.00	0.00	0.00	0.00
GH99	0.00	42.00	23.67	66.00	0.00	51.93	36.12
GH102	0.00	0.00	0.00	231.00	0.00	0.00	0.00
GH103	49.32	0.00	0.00	198.00	0.00	0.00	0.00
GH105	0.00	0.00	1491.25	0.00	0.00	0.00	0.00
GH106/GH43	0.00	0.00	970.50	0.00	0.00	0.00	0.00
GH106/CE15	0.00	0.00	591.77	0.00	0.00	0.00	0.00
GH106	0.00	0.00	1254.54	0.00	0.00	0.00	0.00
GH108	0.00	0.00	0.00	66.00	0.00	0.00	0.00
GH109	0.00	0.00	544.42	0.00	0.00	0.00	0.00
GH110	0.00	0.00	142.02	0.00	0.00	0.00	0.00
GH113	0.00	0.00	0.00	0.00	0.00	0.00	36.12
GH115	0.00	0.00	378.73	0.00	717.59	0.00	0.00
LDA1	0.00	84.00	0.00	0.00	0.00	0.00	0.00
LDA2	0.00	0.00	0.00	0.00	0.00	0.00	108.37
LDA7	0.00	0.00	0.00	99.00	0.00	103.87	0.00
LO1	73.98	0.00	0.00	0.00	0.00	0.00	0.00
LO2	246.61	0.00	0.00	0.00	938.39	0.00	0.00
LO3	49.32	462.01	213.04	560.99	0.00	51.93	252.87

For the analysis, contigs with sizes 1000 bp or larger were used. For each family, the estimated number of nucleotides per million nucleotides was estimated.

The largest number of GHases was found on the contigs assigned to the *Bacteroidetes*, indicating that members of this class are key biomass degraders in the community. This is consistent with their predominant role as biomass decomposers in other biotopes, such as the cow rumen. Also, some key functionalities for the breakdown of hard woods (summarized in Table 4), such as the hydrolysis of (1–2)-α-D-(4-*O*-methyl)glucuronosyl links in the main chain of hardwood xylans by enzymes of the families GH67 and GH115, are only found in contigs assigned to the *Bacteroidetes*. As for lignin breakdown, genes showing homology to LO3 are widespread among the dominant members of the community. However, other putative lignin degrading genes are very patchy: homologs to LO1 multi-copper oxidases were only found among the contigs assigned to the cyanobacteria, whereas the LO2 peroxidase homologs were found on contigs assigned to the cyanobacteria and one cluster of *Clostridiales*.

**Table 4:** Overview of the critical polysaccharide bonds in found in poplar hardwood as well as the glycoside hydrolases that act on these bonds.

Major Polysaccharides	Chemical Linkage Hydrolyzed	General Reaction	EC Name	CAZY GH family
Xylan	D-xylose is β -(1–4) linked to D-xylose in xylan	endohydrolysis of (1–4)-β-D-xylosidic linkages in xylans	endo-β-1,4-xylanaseE.C. 3.2.1.8	5, 8, 10, 11, 43
	D-xylose is β (1–4) linked to D-xylose in xylan	hydrolysis of (1–4)-β-D-xylans to remove successive β-xylose residues from the non-reducing termini	xylan1,4 β-xylosidaseE.C. 3.2.1.37	3, 30, 39, 43, 52, 54, 116, 120
	(*O*-methyl) glucuronosyl is α(1–2) linked to D-xylan in xylan	hydrolysis of (1–2)- α-D-(4-*O*-methyl)glucuronosyl links in the main chain of hardwood xylans	xylan α-1,2-glucuronosidaseE. C. 3.2.1.131	67, 115
Glucomannan	D-mannose is β (1–4) linked to mannose in glucomannan	random hydrolysis of (1–4)- β-D-mannosidic linkages in mannans, galactomannans and glucomannans	mannan endo-1,4-β-mannosidaseE. C. 3.2.1.78	5, 26, 113
	D-mannose is β(1–4) linked to mannose in glucomannan	hydrolysis of (1–4)- β-D-mannosidic linkages in (1–4)- β-D-mannans, removes successive mannobiose residues from the non-reducing chain ends	(exo) mannan 1,4-β- mannobiosidaseE.C. 3.2.1.100	none
	D-mannose is β(1–4) linked to mannose in glucomannan	hydrolysis of terminal, non-reducing β-D-mannosyl residues in β-D-mannosides	β-mannosidaseE. C. 3.2.1.25	1, 2, 5
	D-glucose is β(1–4) linked to D-mannan in glucomannan	hydrolysis of terminal (1–4)-linked α-D-glucosyl residues successively from non-reducing ends of the chains with release of β-D-glucose	β-glucosidaseE.C. 3.2.1.21	1, 3, 9, 30, 116
Cellulose	D-glucose is β(1–4) linked to D-glucose in cellulose	endohydrolysis of (1–4)-β-D-glucosidic linkages in cellulose and cereal β-D-glucans	endo β-1,4-glucanaseE. C. 3.2.1.4	5, 6, 7, 8, 9, 12, 44, 45, 48, 51, 61, 74, 124
	D-glucose is β(1–4) linked to D-glucose in cellulose	hydrolysis of (1–4)-β-D-glucosidic linkages in cellulose and cellotetraose, releasing cellobiose from the non-reducing ends of the chains	(exo) cellulose 1,4-β-cellobiosidaseE.C. 3.2.1.91	5, 6, (7), 9, 48
	D-glucose is β(1–4) linked to D-glucose in cellulose	hydrolysis of terminal (1–4)-linked β-D-glucose residues successively from non-reducing ends of the chains with release of β-D-glucose	β-glucosidaseE. C. 3.2.1.21	1, 3, 9, 30, 116

Minor Polysaccharides	Chemical Linkage	General Reaction	EC Name	CAZY Number
Rhamnoarabinogalactan	D-galactose is β(1–3) linked to D-galactose in rhamnoarabinogalactan	hydrolysis of terminal, non-reducing β-D-galactose residues in (1–3)-β-D-galactopyranans	galactan 1,3-β-galactosidaseE. C. 3.2.1.145	43
	L-rhamnose is α linked to D-galactan in rhamnoarabinogalactan	hydrolysis of terminal α-L-rhamnosyl residues in α-L-rhamnosides	α-L-rhamnosidaseE.C. 3.2.1.40(not known if it acts on 1–6 linkages)	28, 78, 106
	L-arabinose is α(1–6) linked to D-galactan in rhamnoarabinogalactan	hydrolysis of terminal α-L-arabinofuranosyl residues in α-L-arabinosides	α-*N*-arabinofuranosidaseE.C. 3.2.1.55(not known if it acts on α(1–6)	3, 43, 51, 54, 62
Xyloglucan	D-glucose is β-(1–4) linked to D-glucose in xyloglucan	exohydrolysis of 1,4-β-D-glucosidic linkages in xyloglucan	xyloglucan-specific exo - β-1,4-glucanaseE.C. 3.2.1.160 and E.C.3.2.1.155	none
	D-glucose is β-(1–4) linked to D-glucose in xyloglucan	endohydrolysis of 1,4-β-D-glucosidic linkages in xyloglucan	xyloglucan-specific endo-β-1,4-glucanaseE.C. 3.2.1.151	5, 12, 16, 44
	D-glucose is β-(1–4) linked to D-glucose in xyloglucan	hydrolysis of cellobiose from the reducing end of xyloglucans consisting of a (1–4)-β-linked glucan carrying α-D-xylosyl groups on O-6 of the glucose residues	oligoxyloglucan reducing-end-specific cellobiohydrolaseE.C. 3.2.1.150	74
	D-glucose is β-(1–4) linked to D-glucose in xyloglucan	hydrolysis of (1–4)-β-D-glucosidic links in oligoxyloglucans so as to remove successive isoprimeverose [i.e. α-*xylo*-(1–6)-β-D-glucosyl-] residues from the non-reducing ends	oligoxyloglucan β-glycosidaseE.C. 3.2.1.120	none
	D-xylose is α(1–6) linked to D-glucan in xyloglucan	endo hydrolysis of D-xylose α(1–6) linked to glucose	Not found	
	L-fucose is β(1–2) linked to D-xylose side chain of xyloglucan	hydrolysis of terminal disaccharides D-galactosyl L-fucose or D-arabinosyl L-fucose in xylodextrin side chain of xyloglucan	β-D-fucosidaseE.C. 3.2.1.38(not known if acts on these disaccharides)	1, 30
	L-arabinose is α(1–2) linked to D-fucose, which is β(1–2) linked to D-xylose side chain of xyloglucan	hydrolysis of terminal α-L-arabinofuranosyl residues in α-L-arabinosides	α-*N*-arabinofuranosidaseE.C. 2.1.55	3, 43, 51, 54, 62
	D-galactose is α(1–2) linked to D-fucose, which is β 1–2) linked to D-xylose side chain of xyloglucan	hydrolysis of terminal non-reducing β-D-galactofuranosides, releasing galactose	β-galactofuranosidaseE.C. 3.2.1.146	none
Callose	D-glucose is β(1–3) linked to D-glucose in cellulose	hydrolysis of (1–3)-β-D-glucosidic linkages in (1–3)- β-D-glucans	glucan endo-1,3-β-D-glucosidaseE.C. 3.2.1.39	16, 17, 55, 64, 81
	D-glucose is β(1–3) linked to D-glucose in cellulose	successive hydrolysis of β-D-glucosyl residues from the non-reducing ends of (1–3)-β-D-glucans, releasing α-glucose	(exo) glucan 1,3-β-glucosidaseE.C. 3.2.1.58	3, 5, 17, 55
	D-glucose is β(1–3) linked to D-glucose in cellulose	endohydrolysis of (1–3)- or (1–4)-linkages in β-D-glucans when the glucose residue whose reducing group is involved in the linkage to be hydrolysed is itself substituted at C-3	endo-1,3–1,4- β-D-glucanaseE.C. 3.2.1.6	16

Very Minor Polysaccharides	Chemical Linkage	General Reaction	EC Name	CAZY Number
Tension wood Arabinogalactan	D-galactose is β(1–4) linked to D-galactose in arabinogalactan	endohydrolysis of (1–4)-β-D-galactosidic linkages in arabinogalactans	arabinogalactan endo-1,4- β-galactosidaseE.C. 3.2.1.89	53
	D-galactose is β(1–6) linked to galactan in arabinogalactan	endohydrolysis of (1–6)-β-D-galactosidic linkages in arabinogalactan proteins and (1–3):(1–6)-β-galactans to yield galactose and (1–6)-β-galactobiose	galactan endo-1,6-β-galactosidaseE.C. 3.2.1.164	30
	L-arabinose *pyr* is β(1–6) linked to galactan in arabinogalactan	a β-L-arabinoside+H_2_O = L-arabinose+an alcohol	β-L-arabinosidaseE.C. 3.2.1.88	27
	D-glucuronate is β(1–6) linked to D-galactan in arabinogalactan	β-D-glucuronoside+H_2_O = an alcohol+β-D-glucuronate	β-glucuronidaseE.C. 3.2.1.31(not known if acts on 1–6 linkages)	1, 2, 79
	D-galacturonate is β(1–6) linked to D-galactan in arabinogalactan	β-D-galacturonoside+H_2_O = an alcohol+β-D-galacturonate	Not found	
	L-rhamnose is α(1–6) linked to D-galactan in arabinogalactan	hydrolysis of terminal non-reducing α-L-rhamnose residues in α-L-rhamnosides	α-L-rhamnosidasE.C. 3.2.1.40(not known if it acts on 1–6 linkages)	28, 78, 106
	(*O*-methyl) glucuronosyl is α(1–6) linked to galactan in arabinogalactan	hydrolyzes (4-*O*-methyl)glucuronosyl α(1–6) linked to the galactan	Not found	

This database was assembled by first cataloging the known glycosidic bonds in hardwoods (cellulose, hemicelluloses, and pectins) and then correlating reported enzyme activities from the Enzyme Commission (EC) database known or suspected to hydrolyze these bonds [67]–[69]. Finally, the CAZY website database was used to correlate these EC families with the relevant structurally derived glycoside hydrolase (GH) families. “Enzyme Nomenclature", from the “Recommendations of the Nomenclature Committee of the International Union of Biochemistry and Molecular Biology on the Nomenclature and Classification of Enzymes by the Reactions they Catalyse". See http://www.chem.qmul.ac.uk/iubmb/enzyme.

## Discussion

Natural enrichment resulted in a microbial community that degraded poplar under anaerobic conditions, as was evident from strong visual signs of decomposition of the bulk biomass, enzymatic attack of the cell walls, and the disappearance of cellulose and hemi-cellulose. Therefore, this community must contain the necessary functionality to break down the various polymers found in poplar hard wood, including the major polysaccharides xylan and glucomannan as part of the hemicellulose, and cellulose, in addition to less abundant polysaccharides. The complex network of these polysaccharides and their side chains, and the presence of lignin, all contribute to the recalcitrance of the poplar biomass. An overview of the poplar hard wood polysaccharides and the key enzymes that are theoretically required for the efficient decomposition of poplar hard wood are presented in Table 4. When comparing the enzymatic requirements for the breakdown of poplar hardwood with the presence of glycoside hydrolases among the dominant members of the biomass decomposing community (Table 3), most of the necessary activities were found among several of the dominant community members. However, there were some exceptions. For example, hydrolysis of (1–2)-α-D-(4-*O*-methyl)glucuronosyl links in the main chain of hardwood xylans is thought to require enzymes of the GHase Families 67 and 115, both of which were only found among the contigs assigned to *Bacteroides*. And although several other dominant community members encoded putative GHase 43 family members, required for the hydrolysis of terminal non-reducing β-D-galactose residues in (1–3)-β-D-galactopyranans, only *Bacteroides* encoded putative GH43/GH95 and GH95 enzymes (with α-1,2-L-fucosidase and α-L-fucosidase activities). In general, the contigs assigned to *Bacteroides* contained the largest number of unique putative glycoside hydrolases. We therefore hypothesize that the *Bacteroides*-like community members play a major role in the breakdown of cellulose and hemicelluloses under these conditions. Members of this phylum also play an important role in other anaerobic microbiomes with high biomass turnover rates, including the cow rumen [36]. Also, the two *Clostridiales* appear, based on the presence of various putative GHases, to play a key role in the breakdown of these two recalcitrant plant cell wall polysaccharides. This is not unexpected, as many members of the *Clostridiales* have been found in environments with high plant biomass turnover rates.

Because the cellulose and hemicellulose polymers are complexed with lignin, the anaerobic microbes must possess mechanisms to access these polysaccharides, most likely via mechanisms that allow for local depolymerization of lignin, as no major removal of lignin from the cell walls was observed. Despite the lack of enzymes involved in lignin decomposition, the presence of bacterial genes that show homology to fungal lignin oxidases might provide some insights in this process. Homologs to the LO3 family (FOLy database) were common among the six dominant members of the community studied. Most of the genes with similarity to this family are annotated as dihydrolipoamide dehydrogenase, cellobiose dehydrogenases or have more general annotations, as this is a large family of proteins only some of which are involved in lignin breakdown. However, putative cellobiose dehydrogenases can very well be involved in lignin breakdown, as they were found to be able to interact with lignin in three important ways: (1) to break beta-ethers; (2) to demethoxylate aromatic structures in lignins; (3) and to introduce hydroxyl groups in non-phenolic lignins [37], [38]. The presence of LO2 type peroxidases in the *Clostridiales* genome bins might hint towards an anaerobic mechanism for lignin depolymerization, since members of the *Clostridiales* require strict anaerobic conditions for their growth. In addition, genes with homology to the two Dyp-like peroxidase genes, involved in lignin degradation [39] by *Rhodococcus jostii* RHA1, were identified on low-coverage contigs and individual reads, suggesting that minor community members may also be important in local depolymerization of lignin, rendering the hemicellulose and cellulose available to depolymerization by the glycoside hydrolases that are synthesized by many members of the community.

In fungi, the general role of lignin degrading enzymes is perhaps becoming clearer. Vanden-Wymelenberg *et al*. reported up-regulated *Phanerochaete chrysosporium* genes in piles of decaying ball milled pine and the hardwood, aspen [40]. The piles of hardwood (aspen) displayed high transcript levels for a glucose oxidase-like oxidoreductase, a catalase, and an alcohol oxidase [40]. *P. chrysosporium* oxidoreductase transcripts, found when grown on pine, included the copper radical oxidase CRO2 and cellobiose dehydrogenase. In the study presented here, the anaerobic bacterial consortium grown on poplar hardwood appears to produce oxidoreductases more consistent with those produced by *P. chrysosporium* grown on pine, suggesting the bacterial homologs may have different specificity.

Although very little information is available on the anaerobic breakdown of lignin, some genetic clues on the evolution of anaerobic catabolism of aromatic compounds [41], [42] have been described, and anaerobic degradation of aromatic compounds has been reported for various bacteria, including *Azoarcus* spp. CIB [43] and *Geobacter metallireducens* BamVW [44]. Also, members of the genus *Magnetospirillum* that degrade aromatic compounds anaerobically including toluene, phenol and benzoate, were previously isolated from denitrifying enrichment cultures [45], [46]. Although members of this genus have not previously been identified as dominant members of biomass decomposing communities, nor have they been known to harbor metabolic potential to break down lignocellulosic biomass, their unexpected dominant presence in the microbial community decaying poplar biomass seems to indicate that they might play a role in lignin depolymerization or the breakdown of aromatic compounds released from the wood. The toluene-degrading strain *Magnetospirillum* TS-6 was found to contain genes that are homologous to those encoding benzylsuccinate synthase (Bss) and benzoyl-CoA reductase (Bcr), two key enzymes of anaerobic toluene and benzoate degradation respectively in known denitrifying bacteria [45]. These two genes were also found on a large contig that based on sequence composition was assigned to *Magnetospirillum*: a putative *bssD* gene, encoding a benzylsuccinate synthase activating enzyme I, was found immediately downstream of a *bssA* gene coding for a putative alpha subunit of benzylsuccinate synthase (PBDCA2_4994280). Since *Magnetospirillum* does not seem to contain any key functions for lignin depolymerization, such as LO1 and LO2, our hypothesis is that the presence of *Magnetospirillum* is key for the efficient degradation of toxic aromatic compounds that are released from the poplar wood during its decay. As such, *Magnetospirillum* acts as the “liver" of the community, making sure that the community doesn't collapse due to the accumulation of toxic lignin-derived aromatic compounds.

Members of the genus *Magnetospirillum* have been reported to play a significant role in oxic-anoxic transition zones of freshwater ecosystems, where opposing gradients exist of reduced iron and sulfide with oxygen, creating a suitable environment for microorganisms that derive energy from the oxidation of iron or sulfide [47]. It cannot be totally excluded that opposing redox gradient also existed in our reactor system, and that this resulted in the enrichment of *Magnetospirillum*.

The distribution and relative abundance of cellulases, hemicellulases, debranching enzymes and enzymes homologous to lignolytic enzymes revealed compelling patterns of enzyme frequencies among different types of communities. In this respect, the strong conservation in the relative distribution of candidate GHase between the top and bottom layers of the fungal garden, as shown in Figure S3, is striking, as both layers have a clear distinction: the top layer, which retains the green, harvested state of plant leaves; and the bottom layer, which contains mature fungus and partially degraded plant material. Furthermore, comparison of the phylogenetic diversity between top and bottom layer indicated distinct differences, although both layers are dominated by phylotypes in the α-*Proteobacteria*, β-*Proteobacteria*, γ-*Proteobacteria*, *Actinobacteria* and *Bacteroidetes* [30]. The common presence of these dominant phylotypes might explain the conservation in functionality. It was also noted that no measurable lignin degradation occurred in the fungal garden. However, in order to explain the observed breakdown of hemicellulose and cellulose, some local degradation of lignin is required in order to access these polymers. The presence of bacterial genes with similarity to fungal peroxidases might provide a possibility for lignin breakdown.

In comparison, more light has been shed recently regarding the complete picture of fungal cellulose degradation by the discovery of the probable role of GH Family 61 oxidative enzymes [48]. For example, Vanden-Wymelenberg *et al.* found high levels of GH61 transcripts in decaying softwood piles inoculated with *P. chrysosporium* [40]. In the study presented here, the anaerobic bacterial consortium grown on the hardwood, poplar, appears to lack the ability to produce GH61 enzymes. Recently, the cellulose binding module (CBM) from Family 33, which is a structural analogue to GH61, was implicated in bacterial oxidative cellulose deconstruction [49]. In this consortium, four homologs of CBM33 were found, all located on relatively small unbinned sequences: two on individual 454 reads, and 2 on contigs of 2–3× depth. All putative CBM33 genes showed high GC content (64–69%), so on that basis could potentially belong to the *Magnetospirillum*, whose contigs varied considerably in coverage due to apparent sequencing bias in this high-GC genome. Furthermore, the bacterial consortium studied here encodes putative cellobiose dehydrogenases. This is significant, because it was suggested nearly 15 years ago that cellobiose dehydrogenase could be involved in cellulose depolymerization [50] and today it appears that GH61 (or CBM33) and cellobiose dehydrogenase could work cooperatively to effect oxidative cellulose depolymerization.

From a community perspective, a striking observation is the near-complete lack of putative lignin degrading enzymes shared by all the gut communities examined (termite hindgut, cow rumen, wallaby, dog, human and mouse); whereas compost, soil, and fungal garden microbes all harbored some of these genes, as did the members of the poplar biomass decomposing community. For example, the termite hindgut community has very few candidate functions involved in the decomposition of lignin; only one candidate LDA6 with homology to a glucose oxidase and 20 putative LO3 candidates, all displaying homology to dihydrolipoyl and dihydrolipoamide dehydrogenases, could be identified (Fig. 7C). The lack of enzymatic functions involved in the decomposition of lignin in the gut communities suggests that the hosts may provide adequate pretreatment of the biomass to allow for microbial decomposition, through chewing in all animals in addition to an alkaline pretreatment in the higher termite gut (pH 8.5–12, depending on species) [51], [52], which might provide suitable solubilization of some lignins to permit the needed GHase accessibility to wall polysaccharides. While the relative availability of oxygen may also impact on the prevalence of lignin degradation pathways, it is worth noting that the termite gut has been found to have relatively high oxygen concentrations in some areas [53]. Significant lignin degradation has been proposed to occur in the Asian longhorned beetle, which could prove an exception to the pattern seen here [54]. This near-complete lack of putative lignin degrading enzymes was shared by all other gut communities examined (including bovine rumen, wallaby, dog, human and mouse). In the case of the termite gut, the relatively high pH (10–12) of the higher gut may provide suitable solubilization of some lignins to permit the needed GHase accessibility to wall polysaccharides.

In conclusion, natural communities subsisting on untreated plant biomass provide an ideal environment for the bioprospecting of enzymes involved in the depolymerization of plant cell-wall polysaccharides and lignin. Our results seem to indicate that these communities have a much broader metabolic potential than host-associated communities and thus could provide a richer resource for finding new catalytic functions involved in biomass decomposition. This is exemplified by the microbial community that under anaerobic conditions is decaying poplar woodchips, as a very broad representation of glycoside hydrolases and putative lignin decomposing enzymes was found. Furthermore we unexpectedly identified bacteria among the dominant community members, similar to *Magnetospirillum*, that seem to play a key role in the anaerobic breakdown of aromatic compounds. We hypothesize that these compounds are released from the lignin fraction in the poplar hardwood during the decay process, which would point to lignin-depolymerization under anaerobic conditions.

## Materials and Methods

### Preparation of biomass and DNA samples

This work concentrated on the microbial community decaying poplar biomass under anaerobic conditions. 1.8 kg non-sterile yellow poplar saw dust, with particles ranging in sizes between 1 mm^3^ to 0.3 cm^3^, was taken from the inside of a 1 m^3^ pile and placed in a plastic, white, 10 L bucket. The biomass was humidified with 5 L 10 mM MgSO_4_ solution and the bucket was closed with an air-tight plastic cover and incubated at 30°C. This temperature was chosen over thermophilic conditions, as we expected that the organisms naturally present on the biomass were unlikely to be adapted to thermophilic conditions. This resulted in the creation of a gradient ranging from micro aerobic at the top to anaerobic at the bottom of the biomass. After 3 and 12 months incubation in the dark at 30°C, 500 g biomass and 500 mL liquid were collected from the anaerobic zone at the bottom of the bucket and used for DNA isolation and imaging studies. In addition, a sample was taken from the aerobic zone for imaging studies.

The attached microorganisms were suspended in 10 mM MgSO_4_ solution by vigorously shaking the biomass, and collected by centrifugation, after which total community DNA was isolated according to Bron and Venema [55]. To further purify the DNA, the material was loaded on a 0.8% agarose gel, and after migration DNA with a size of >23 kb was isolated using the QIAEX II Gel Extraction Kit (Qiagen, Valencia, CA, USA) according to the manufacturer's instructions. The DNA isolated with this method had a molecular weight of over 40 kb and was suitable for PCR amplification and restriction digestion.

### Sample Preparation for Microscopy

Pieces of untreated and composted poplar tissues were fixed and embedded using microwave processing. Samples were fixed 2×6 min in 2.5% gluteraldehyde buffered in 0.1 M sodium cacodylate buffer (EMS, Hatfield, PS) under vacuum. The samples were dehydrated by treating with increasing concentrations of ethanol for 1 min at each dilution (30%, 60%, 90%, and 3× 100% ethanol). The samples were infiltrated with Epon resin (EMS, Hatfield, PA) for 3 min, with one final step at room temperature (RT) overnight, in increasing concentrations of resin (7%, 15%, 30%, 60%, 90%, 3× 100% resin, diluted in ethanol). Infiltrated samples were transferred to flat-bottomed TAAB capsules and polymerized at 60°C for 24 h. Epon-embedded samples were sectioned to 2 µm with glass knives on a Leica EM UTC ultramicrotome (Leica, Wetzlar, Germany) for confocal scanning laser microscopy (CSLM) and epifluorescent light microscopy (ELM) or sectioned to 60 nm with a Diatome diamond knife on a Leica EM UTC ultramicrotome (Leica, Wetzlar, Germany) for transmission electron microscopy and Coherent anti-Stokes Raman (CARS) microscopy.

### Transmission Electron Microscopy

Ultra-thin sections were collected on formvar coated copper slot grids (SPI Supplies, West Chester, PA). Grids were post-stained for 6 min with 2% aqueous uranyl acetate and 3 min with Reynolds lead citrate. Samples were finally analyzed on a FEI Tecnai G2 20 Twin 200 kV LaB6 TEM (FEI, Hilsboro, OR) equipped with a 4 mega-pixel Gatan UltraScan 1000 camera (Gatan, Pleasanton, CA).

### Coherent anti-Stokes Raman (CARS) microscopy

CARS microscopy [56], [57] was used to provide information on biomass composition. A mode-locked Nd∶VAN laser (High Q Laser (US), Inc) was used to generate 7 ps, 76 MHz pulse trains of both a 1064 nm and 532 nm laser beam. The 1064 nm beam was used as the Stokes beam. The 532 nm beam was used to pump an optical parametric oscillator (OPO) (Levante Emerald, APE-Berlin) to generate the CARS pump beam. The pump beam was tuned to 910 nm to effectively detect the 1600 cm^−1^ Raman band. The excitation power was kept at ∼350 mW for the pump and ∼150 mW for the Stokes beam. Samples were cut into ∼20 µm thin sections by rotary microtome (Leica RM2255, Leica Microsystems Inc) embedding processes, after which slices were spread out between two cover slips. CARS imaging of the lignin was obtained at 1600 cm^−1^. The focused beams were raster-scanned over the sample. The anti-Stokes light was collected from the Epi-direction and filtered by a clean-up filter 800/40 (Thorlabs).

### Microbial cultivation

In order to isolate members of the genus *Clostridium*, dilutions of the microbial suspension obtained from the anaerobic zone were plated on CM3 medium [58] containing cellulose, hemicellulose, cellobiose or glucose and incubated under anaerobic conditions at 30°C.

### DNA sequencing

To obtain a first glance of the community composition after 12 months of incubation, DNA was extracted from the bioreactor material and was used for construction of a bacterial 16 S clone library (see http://my.jgi.doe.gov/general/protocols/), from which 384 clones were sequenced. 16 S rRNA gene sequences were submitted to Genbank and assigned accession numbers JQ624944 to JQ625053. Subsequently, the DNA subjected to extensive 16 S/18 S profiling by V6–V8 pyrotag sequencing using the primers 926F and 1391R, reported to be capable of amplifying bacteria, fungi, and some archaea [59]. A total of 2,549,998 sequencing reads were generated and analyzed with the in-house pipeline PyroTagger [60], which filters out low-quality or short sequences, trims to a uniform length, clusters at 97% identity and blasts cluster representatives against a combined Greengenes [61]/Silva [62] database of aligned rRNA sequences from the *Bacteria, Archaea* and *Eukarya* domains. In total 1,516,612 16 S/181S reads were clustered and phylogenetically assigned. The pyrotag data were submitted to the Short Read Archive under SRA number SRA045915.

The same bioreactor DNA used for community profiling was used to construct both a short-insert (3 kb) and a fosmid (40 kb) metagenome library for Sanger end sequencing. 11 Mb and 31 Mb of sequence were generated from these libraries, respectively. To obtain much deeper coverage of the constituent species, the Sanger data were supplemented with 454 pyrosequencing data, both using the FLX technology (115 Mb from a full run) and Titanium (564 Mb from a full run). Finally, to improve scaffolding another half Titanium run (236 Mb) was sequenced from a 454 paired-end library with a 3 kb insert size. All the resulting data were trimmed with the program LUCY [63] to remove low quality, vector, and adapter sequence and de-replicated with Uclust (http://drive5.com/usearch/usearch3.0.html) to remove duplicate reads prior to assembly. Data were assembled using Newbler 2.4. The pyrotag data were submitted to the Short Read Archive under SRA number SRA045915. The metagenome data, including all assembled gene sequences, can be publically accessed via the IMG/M website at http://img.jgi.doe.gov/cgi-bin/m/main.cgi?section=TaxonDetail&taxon_oid=2010388001, and via GenBank accession number AGTN00000000.1. In addition, for each of the assembled glycoside hydrolase or lignin oxidase genes, their IMG/M and CAZY/FOLY identifiers were provided as part of the Supporting Information as File S1, 'GHases and LO overview.xls.

### Metagenome annotation, analysis and binning

All contigs and unassembled singlets were submitted to IMG/M-ER for annotation [64]. Phylogenetically informative genes in the metagenome were identified with the “Phylogenetic Marker COGs" function and used to build trees with homologs from isolate genomes and thereby identify contigs and scaffolds belonging to the dominant populations. Contigs and scaffolds from six abundant populations (*Magnetospirillum*, *Bacteroides*, *Methanomicrobia*, *Cyanobacteria*, and two *Clostridiales*) were used as training data for the metagenome classifier, ClaMS [65]. The classifier was then used to bin all remaining contigs >2 kb, of which ∼63% were assigned to one of the populations. These bins were then added to the dataset in IMG/M-ER.

All metagenome scaffold sequences used here were obtained from IMG/M [64]. For each metagenome, including the poplar biomass bioreactor, scaffold sequences were divided into 300 nt fragments (tiles) overlapping by 150 nt. BlastX [66] searches of each fragment were then conducted vs. the complete set of protein sequences from the CAZy (http://www.cazy.org/) [21] and FOLy (http://foly.esil.univ-mrs.fr) [22] websites. Contiguous runs of best hits with e-value below a 1e-10 threshold and having the same CAZy or FOLy gene family were collected from each fragment. These run lengths were then multiplied by mean contig depth and totaled by family. A depth of 1 was assumed for those metagenomes for which contig depth information was not available.

Glycoside hydrolase and ligninolytic metagenome comparison scatterplots were made by normalizing these family totals by metagenome total sequence, weighted by mean contig depth. Clustering and MDS plots comparing the metagenomes were created from the normalized GH values using Spearman rank correlation coefficients for distance/similarity. BlastX batch jobs were run in parallel on a 40 CPU linux cluster using PBS/Torque. Post-processing was performed with a combination in-house Perl and R scripts. Plots were created with R.

## Supporting Information

Figure S1 **Stereo light micrographs of control (A) and composted (B) poplar particles.** The composted particles were darker and softer, but remained intact. Scale bars = 1 mm.
(TIF)

Figure S2 **Confocal scanning laser micrographs of control (A) and composted (B) poplar tissues.** The green fluorescent signal from the LM11::Alexa 488 anti-xylan antibody displays a fairly uniform distribution with higher concentration in the compound middle lamella (arrow) in control samples (A). The xylan distribution in composted samples appears patchy (B, arrows) within disrupted, thinner walls. Scale bars = 100 µm.
(TIF)

Figure S3 **Comparison of the total coverage per family of biomass modifying enzymes for the metagenomes of the top versus bottom of the fungal garden.** This comparison includes cellulases, hemicellulases, debranching enzymes and enzymes homologous to lignolytic enzymes.
(TIF)

File S1 **GHases and LO overview.** IMG/M and CAZY/FOLY identifiers for putative glycoside hydrolases and lignin oxidizing enzymes identified among the metagenome of the microbial community decaying poplar wood chips.
(XLS)

Methods S1 **Supplemental materials and methods.**
(DOCX)

## References

[1] Himmel ME, Himmel ME (2008). Biomass recalcitrance - deconstructing the plant cell wall for bioenergy.

[2] Bayer EA, Belaich J-P, Shoham Y, Lamed R (2004). THE CELLULOSOMES: Multienzyme Machines for Degradation of Plant Cell Wall Polysaccharides.. Annual Rev Microbiol.

[3] Bayer EA, Shimon LJ, Shoham Y, Lamed R (1998). Cellulosomes-structure and ultrastructure..

[4] Himmel ME, Saha BC, Woodward J (1997). Fuels and Chemicals from Biomass..

[5] Davies G, Henrissat B (1995). Structures and mechanisms of glycosyl hydrolases.. Structure.

[6] Huang DL, Zeng GM, Feng CL, Hu S, Lai C (2010). Changes of microbial population structure related to lignin degradation during lignocellulosic waste composting.. Bioresour Technol.

[7] Yu H, Zeng G, Huang H, Xi X, Wang R (2007). Microbial community succession and lignocellulose degradation during agricultural waste composting.. Biodegradation.

[8] Kshattriya S, Jha DK, Sharma GD, Mishra RR (1996). Litter decomposition in relation to soil nitrogen dynamics in two degraded tropical forest stands.. Ecoprint.

[9] Majumder M, Shukla AK, Arunachalam A (2008). Nutrient release and fungal succession during decomposition of weed residues in a shifting cultivation system.. Communications in Biometry and Crop Science.

[10] Amann RJ, Binder BL, Chisholm SW, Devereux R, Stahl DA (1990). Combination of 16 S rRNA targeted oligonucleotide probes with flow-cemetry for analysing mixed microbial populations.. Appl Environ Microbiol.

[11] Daniel R (2005). The metagenomics of soil.. Nat Rev Microbiol.

[12] Lorenz P, Eck J (2005). Metagenomics and industrial applications.. Nature.

[13] Li LL, McCorkle SM, Monchy S, Taghavi S, van der Lelie D (2009). Bioprospecting metagenomes: glycosyl hydrolases for converting biomass.. Biotechnol Biofuels.

[14] Tyson GW, Chapman J, Hugenholtz P, Allen EE, Ram RJ (2004). Community structure and metabolism through reconstruction of microbial genomes from the environment.. Nature.

[15] Gilbert JA, Meyer F, Bailey MJ (2011). The Future of microbial metagenomics (or is ignorance bliss?).. ISME J.

[16] Heidelberg KB, Gilbert JA, Joint I (2010). Marine genomics: at the interface of marine microbial ecology and biodiscovery.. Microb Biotechnol.

[17] Gilbert JA, Dupont CL (2011). Microbial metagenomics: beyond the genome.. Ann Rev Mar Sci.

[18] Hess M, Sczyrba A, Egan R, Kim TW, Chokhawala H (2011). Metagenomic discovery of biomass-degrading genes and genomes from cow rumen.. Science.

[19] Miller JR, Koren S, Sutton G (2010). Assembly algorithms for next-generation sequencing data.. Genomics.

[20] Schluter A, Bekel T, Diaz NN, Dondrup M, Eichenlaub R (2008). The metagenome of a biogas-producing microbial community of a production-scale biogas plant fermenter analysed by the 454-pyrosequencing technology.. J Biotechnol.

[21] Cantarel BL, Coutinho PM, Rancurel C, Bernard T, Lombard V (2009). The Carbohydrate-Active EnZymes database (CAZy): an expert resource for Glycogenomics.. Nucleic Acids Res.

[22] Levasseur A, Piumi F, Coutinho PM, Rancurel C, Asther M (2008). FOLy: an integrated database for the classification and functional annotation of fungal oxidoreductases potentially involved in the degradation of lignin and related aromatic compounds.. Fungal Genet Biol.

[23] Lairson LL, Henrissat B, Davies GJ, Withers SG (2008). Glycosyltransferases: structures, functions, and mechanisms.. Annu Rev Biochem.

[24] Erb A, Weiss H, Harle J, Bechthold A (2009). A bacterial glycosyltransferase gene toolbox: generation and applications.. Phytochemistry.

[25] Stam MR, Danchin EG, Rancurel C, Coutinho PM, Henrissat B (2006). Dividing the large glycoside hydrolase family 13 into subfamilies: towards improved functional annotations of alpha-amylase-related proteins.. Protein Eng Des Sel.

[26] Harvey AJ, Hrmova M, De Gori R, Varghese JN, Fincher GB (2000). Comparative modeling of the three-dimensional structures of family 3 glycoside hydrolases.. Proteins.

[27] Lee RC, Hrmova M, Burton RA, Lahnstein J, Fincher GB (2003). Bifunctional family 3 glycoside hydrolases from barley with alpha -L-arabinofuranosidase and beta -D-xylosidase activity. Characterization, primary structures, and COOH-terminal processing.. J Biol Chem.

[28] Li LL, Taghavi S, McCorkle SM, Zhang YB, Blewitt MG (2011). Bioprospecting metagenomics of decaying wood: mining for new glycoside hydrolases.. Biotechnol Biofuels.

[29] Allgaier M, Reddy A, Park JI, Ivanova N, D'Haeseleer P (2010). Targeted discovery of glycoside hydrolases from a switchgrass-adapted compost community.. PLoS One.

[30] Suen G, Scott JJ, Aylward FO, Adams SM, Tringe SG (2010). An insect herbivore microbiome with high plant biomass-degrading capacity.. PLoS Genet.

[31] Warnecke F, Luginbuhl P, Ivanova N, Ghassemian M, Richardson TH (2007). Metagenomic and functional analysis of hindgut microbiota of a wood-feeding higher termite.. Nature.

[32] Pope PB, Denman SE, Jones M, Tringe SG, Barry K (2010). Adaptation to herbivory by the Tammar wallaby includes bacterial and glycoside hydrolase profiles different from other herbivores.. Proc Natl Acad Sci U S A.

[33] Swanson KS, Dowd SE, Suchodolski JS, Middelbos IS, Vester BM (2011). Phylogenetic and gene-centric metagenomics of the canine intestinal microbiome reveals similarities with humans and mice.. ISME J.

[34] Gill SR, Pop M, Deboy RT, Eckburg PB, Turnbaugh PJ (2006). Metagenomic analysis of the human distal gut microbiome.. Science.

[35] Turnbaugh PJ, Ley RE, Mahowald MA, Magrini V, Mardis ER (2006). An obesity-associated gut microbiome with increased capacity for energy harvest.. Nature.

[36] Ramsak A, Peterka M, Tajima K, Martin JC, Wood J (2000). Unravelling the genetic diversity of ruminal bacteria belonging to the CFB phylum.. FEMS Microbiol Ecol.

[37] Henriksson G, Zhang L, Li J, Ljungquist P, Reitberger T (2000). Is cellobiose dehydrogenase from Phanerochaete chrysosporium a lignin degrading enzyme?. Biochim Biophys Acta.

[38] Hilden L, Johansson G, Pettersson G, Li J, Ljungquist P (2000). Do the extracellular enzymes cellobiose dehydrogenase and manganese peroxidase form a pathway in lignin biodegradation?. FEBS Lett.

[39] Ahmad M, Roberts JN, Hardiman EM, Singh R, Eltis LD (2011). Identification of DypB from Rhodococcus jostii RHA1 as a Lignin Peroxidase.. Biochemistry.

[40] Vanden Wymelenberg A, Gaskell J, Mozuch M, Splinter Bondurant S, Sabat G (2011). Significant Alteration of Gene Expression in Wood Decay Fungi Postia placenta and Phanerochaete chrysosporium by Plant Species.. Appl Environ Microbiol.

[41] Barragan MJL, Diaz E, Garcia JL, Carmona M (2004). Genetic clues on the evolution of anaerobic catabolism of aromatic compounds.. Microbiology-Sgm.

[42] Carmona M, Zamarro MT, Blazquez B, Durante-Rodriguez G, Juarez JF (2009). Anaerobic Catabolism of Aromatic Compounds: a Genetic and Genomic View.. Microbiol Mol Biol Rev.

[43] Molina MTZ, Carmona M, Barragan MJL, Blaquez B, Garcia JL (2009). Genome mining in Azoarcus spp. CIB: a model bacterium to engineer biocatalysts for anaerobic removal of aromatic compounds.. New Biotechnol.

[44] Juarez JF, Zamarro MT, Barragan MJL, Blazquez B, Boll M (2010). Identification of the Geobacter metallireducens BamVW Two-Component System, Involved in Transcriptional Regulation of Aromatic Degradation.. Appl Env Microbiol.

[45] Shinoda Y, Akagi J, Uchihashi Y, Hiraishi A, Yukawa H (2005). Anaerobic degradation of aromatic compounds by Magnetospirillum strains: Isolation and degradation genes.. Biosci Biotechnol Biochem.

[46] Shinoda Y, Sakai Y, Ue M, Hiraishi A, Kato N (2000). Isolation and characterization of a new denitrifying spirillum capable of anaerobic degradation of phenol.. Appl Environ Microbiol.

[47] Geelhoed JS, Sorokin DY, Epping E, Tourova TP, Banciu HL (2009). Microbial sulfide oxidation in the oxic-anoxic transition zone of freshwater sediment: involvement of lithoautotrophic Magnetospirillum strain J10.. FEMS Microbiol Ecol.

[48] Harris PV, Welner D, McFarland KC, Re E, Navarro Poulsen JC (2010). Stimulation of lignocellulosic biomass hydrolysis by proteins of glycoside hydrolase family 61: structure and function of a large, enigmatic family.. Biochemistry.

[49] Vaaje-Kolstad G, Westereng B, Horn SJ, Liu Z, Zhai H (2010). An oxidative enzyme boosting the enzymatic conversion of recalcitrant polysaccharides.. Science.

[50] Mansfield SD, De Jong E, Saddler JN (1997). Cellobiose dehydrogenase, an active agent in cellulose depolymerization.. Appl Environ Microbiol.

[51] Thongaram T, Hongoh Y, Kosono S, Ohkuma M, Trakulnaleamsai S (2005). Comparison of bacterial communities in the alkaline gut segment among various species of higher termites.. Extremophiles.

[52] Bignell DE, Eggleton P (1995). On the elevated gut pH of higher termites (Isoptera: Termitidae).. Insectes Sociaux.

[53] Brune A, Emerson D, Breznak JA (1995). The Termite Gut Microflora as an Oxygen Sink: Microelectrode Determination of Oxygen and pH Gradients in Guts of Lower and Higher Termites.. Appl Environ Microbiol.

[54] Geib SM, Filley TR, Hatcher PG, Hoover K, Carlson JE (2008). Lignin degradation in wood-feeding insects.. Proc Natl Acad Sci U S A.

[55] Bron S, Venema G (1972). Ultraviolet inactivation and excision-repair in Bacillus subtilis. IV. Integration and repair of ultraviolet-inactivated transforming DNA.. Mutat Res.

[56] Saar BG, Zeng YN, Freudiger CW, Liu YS, Himmel ME (2010). Label-Free, Real-Time Monitoring of Biomass Processing with Stimulated Raman Scattering Microscopy.. Angewandte Chemie-International Edition.

[57] Zeng YN, Saar BG, Friedrich MG, Chen F, Liu YS (2010). Imaging Lignin-Downregulated Alfalfa Using Coherent Anti-Stokes Raman Scattering Microscopy.. Bioenergy Research.

[58] Weimer PJ, Zeikus JG (1977). Fermentation of cellulose and cellobiose by Clostridium thermocellum in the absence of Methanobacterium thermoautotrophicum.. Appl Environ Microbiol.

[59] Engelbrektson A, Kunin V, Wrighton KC, Zvenigorodsky N, Chen F (2010). Experimental factors affecting PCR-based estimates of microbial species richness and evenness.. ISME J.

[60] Kunin V, Hugenholtz P (2010). PyroTagger: A fast, accurate pipeline for analysis of rRNA amplicon pyrosequence data.. The Open Journal.

[61] DeSantis TZ, Hugenholtz P, Larsen N, Rojas M, Brodie EL (2006). Greengenes, a chimera-checked 16 S rRNA gene database and workbench compatible with ARB.. Appl Environ Microbiol.

[62] Pruesse E, Quast C, Knittel K, Fuchs BM, Ludwig W (2007). SILVA: a comprehensive online resource for quality checked and aligned ribosomal RNA sequence data compatible with ARB.. Nucleic Acids Res.

[63] Chou HH, Holmes MH (2001). DNA sequence quality trimming and vector removal.. Bioinformatics.

[64] Markowitz VM, Chen IM, Palaniappan K, Chu K, Szeto E (2010). The integrated microbial genomes system: an expanding comparative analysis resource.. Nucleic Acids Res.

[65] Pati A, Heath LS, Kyrpides NC, Ivanova N (2011). ClaMS: A Classifier for Metagenomic Sequences.. Stand Genomic Sci.

[66] Camacho C, Coulouris G, Avagyan V, Ma N, Papadopoulos J (2009). BLAST+: architecture and applications.. BMC Bioinformatics.

[67] Decou R, Lhernould S, Laurans F, Sulpice E, Leple JC (2009). Cloning and expression analysis of a wood-associated xylosidase gene (PtaBXL1) in poplar tension wood.. Phytochem.

[68] Laine C (2005).

[69] Sjostrom J (1993). Wood Chemistry, Fundamentals and Applications. 2nd edition.

