# Supplementary figures and images for: The Metagenome of an Anaerobic Microbial Community Decomposing Poplar Wood Chips
Source: PLoS One. 2012 May 21;7(5):e36740. doi: 10.1371/journal.pone.0036740 (PMC3357426; doi:10.1371/journal.pone.0036740)

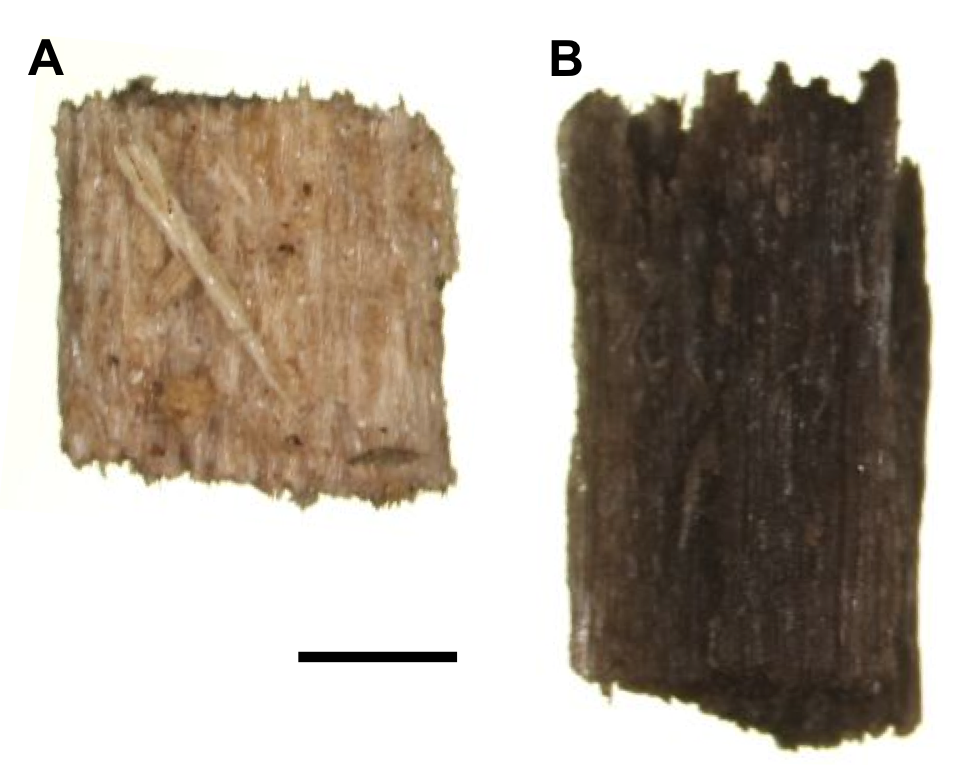

Supplement: Figure S1 — Stereo light micrographs of control (A) and composted (B) poplar particles. The composted particles were darker and softer, but remained intact. Scale bars = 1 mm. (TIF) [file pone.0036740.s001.tif]

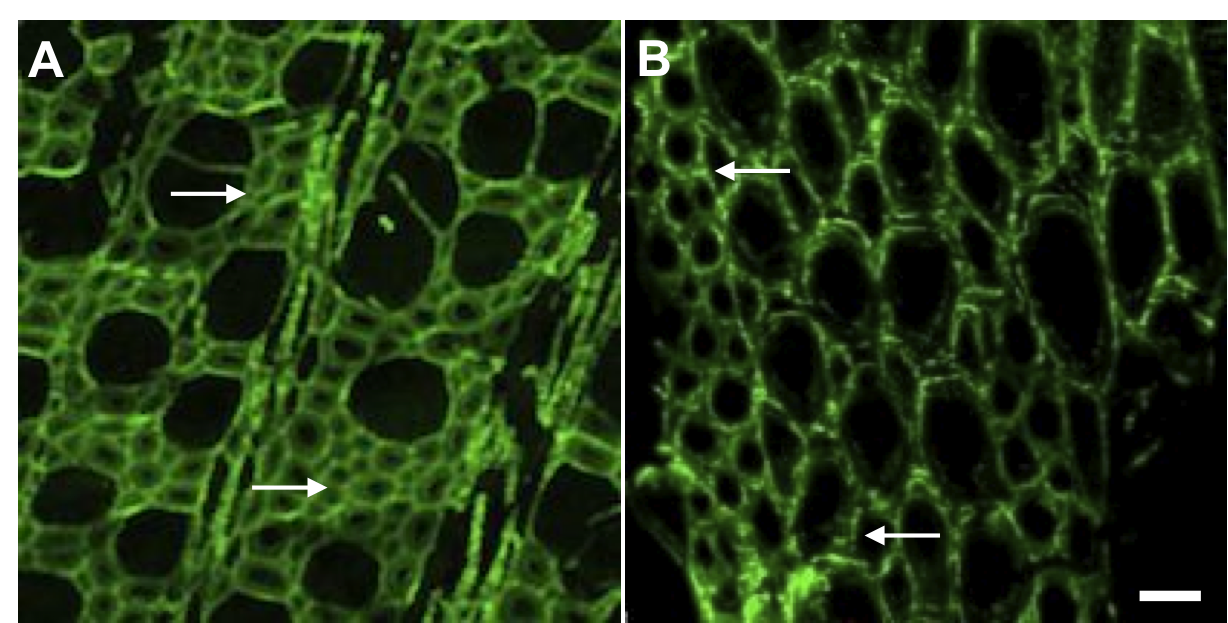

Supplement: Figure S2 — Confocal scanning laser micrographs of control (A) and composted (B) poplar tissues. The green fluorescent signal from the LM11::Alexa 488 anti-xylan antibody displays a fairly uniform distribution with higher concentration in the compound middle lamella (arrow) in control samples (A). The xylan distribution in composted samples appears patchy (B, arrows) within disrupted, thinner walls. Scale bars = 100 µm. (TIF) [file pone.0036740.s002.tif]

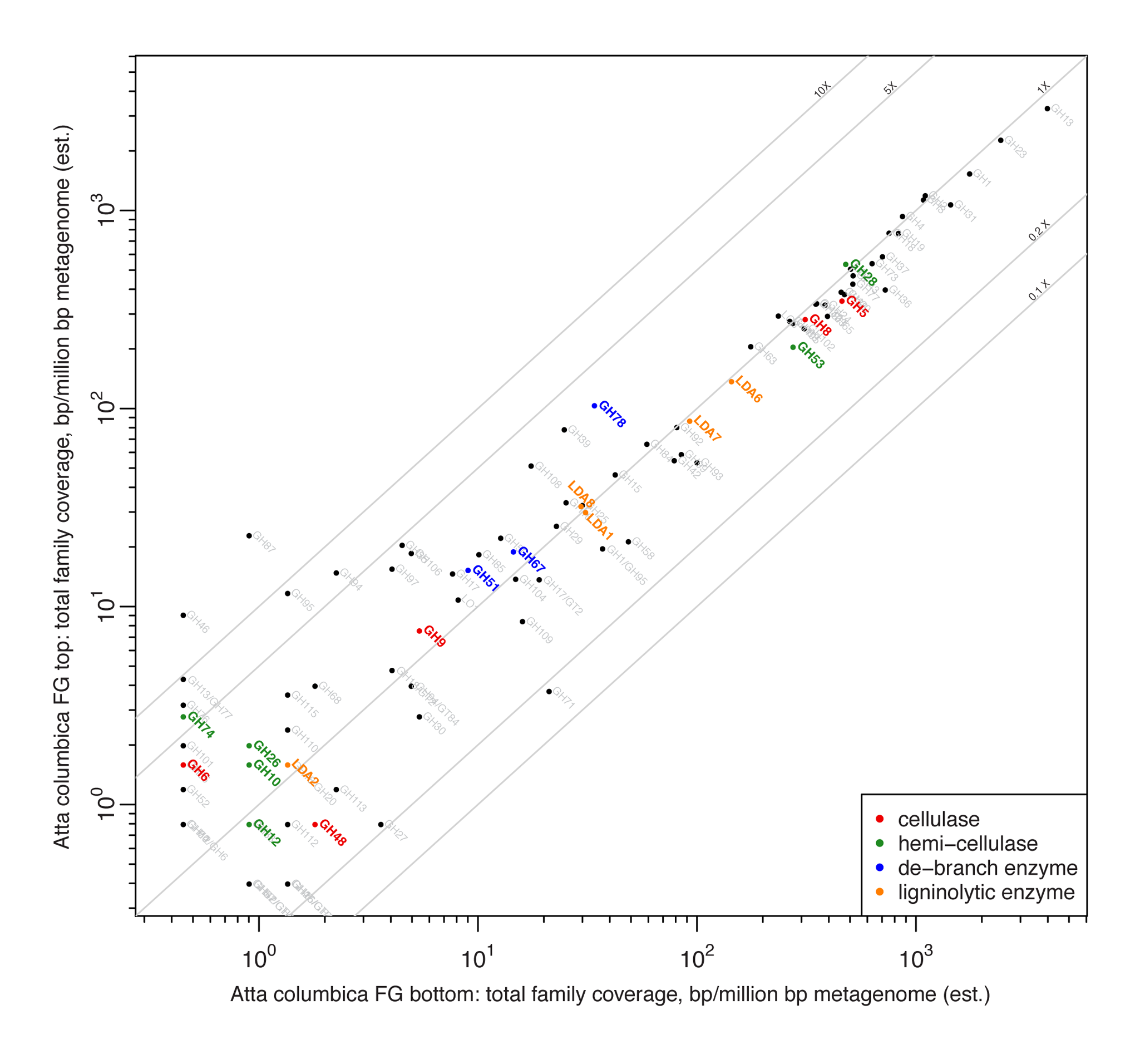

Supplement: Figure S3 — Comparison of the total coverage per family of biomass modifying enzymes for the metagenomes of the top versus bottom of the fungal garden. This comparison includes cellulases, hemicellulases, debranching enzymes and enzymes homologous to lignolytic enzymes. (TIF) [file pone.0036740.s003.tif]
