# Supplementary material for: The Metagenome of an Anaerobic Microbial Community Decomposing Poplar Wood Chips
Source: PLoS One. 2012 May 21;7(5):e36740. doi: 10.1371/journal.pone.0036740 (PMC3357426; doi:10.1371/journal.pone.0036740)
Supplement: Methods S1 — Supplemental materials and methods. (DOCX) [file pone.0036740.s005.docx]

**The metagenome of an anaerobic microbial community decomposing poplar wood chips**

**Daniel van der Lelie^1,2,3*^, Safiyh Taghavi^1,2^, Sean M. McCorkle^1,2^, Luen-Luen Li^1,2^, Stephanie A. Malfatti^4^, Denise Monteleone^1,2^, Bryon S. Donohoe^2,5^, Shi-You Ding^2,5^, William S. Adney^2,5^, Michael E. Himmel^2,5^, and Susannah G. Tringe^4^**

^1^ Brookhaven National Laboratory, Biology Department, 50 Bell Avenue, Upton, NY11973-5000, USA

^2^ BioEnergy Science Center, Oak Ridge National Laboratory, Oak Ridge, TN37831, USA

^3^RTI International, Center for Agricultural and Environmental Biotechnology, 3040 Cornwallis Road Research, Triangle Park, NC27709, USA

^4^ DOE Joint Genome Institute, 2800 Mitchell Drive, Walnut Creek, CA94598, USA

^5^ National Renewable Energy Laboratory, 16253 Denver West Parkway, Golden, CO 80401, USA

* Correspondence should be addressed to DvdL:

E-mail: [vdlelied@rti.org](mailto:vdlelied@rti.org); Phone: +1-919.316.3532

**Supplemental Materials and Methods**

***Sample Preparation for Microscopy***

Pieces of untreated and composted poplar tissues were fixed and embedded using microwave processing. Samples were fixed 2 x 6 min in 2.5% gluteraldehyde buffered in 0.1 M sodium cacodylate buffer (EMS, Hatfield, PS) under vacuum. The samples were dehydrated by treating with increasing concentrations of ethanol for 1 min at each dilution (30%, 60%, 90%, and 3X 100% ethanol). The samples were infiltrated with Epon resin (EMS, Hatfield, PA) for 3 min, with one final step at room temperature (RT) overnight, in increasing concentrations of resin (7%, 15%, 30%, 60%, 90%, 3X 100% resin, diluted in ethanol). Infiltrated samples were transferred to flat-bottomed TAAB capsules and polymerized at 60°C for 24 h. Epon-embedded samples were sectioned to 2 µm with glass knives on a Leica EM UTC ultramicrotome (Leica, Wetzlar, Germany) for confocal scanning laser microscopy (CSLM) and epifluorescent light microscopy (ELM) or sectioned to 60 nm with a Diatome diamond knife on a Leica EM UTC ultramicrotome (Leica, Wetzlar, Germany) for transmission electron microscopy and Coherent anti-Stokes Raman (CARS) microscopy.

***Immuno-labeling***

For ELM and CSLM of embedded untreated and composted poplar tissues, sections were placed on ProbeOn Plus (Fisher Scientific, Pittsburgh, PA) microscope slides and incubated in 5% non-fat dry milk w/v PBS-0.1% Tween 20 (milk/PBST) blocking solution for 30 min at 25ºC. Primary probes: PentaHIS-CBM3 (40 µg/mL milk/PBST) (Qi Xu, NREL), rat α-pectin JIM5 (1:5 v/v milk/PBST dilution) (Carbosource, Athens, GA), and/or rat α-xylan LM11; were applied on sections for 1.5 h at 25ºC and then rinsed 3x with PBST. Secondary probes: α-PentaHIS: Alexa555 (against CBM3, 1:50 milk/PBST dilution) (Qiagen, Hilden, Germany), goat α-rat IgG::Alexa488 (against JIM5, 1:200 milk/PBST dilution) and goat α-rat IgG::Alexa488 (against LM11, 1:200 milk/PBST dilution) (Molecular Probes, Eugene, OR); were applied on sections for 1.5 h and then rinsed three times with PBST. Sections were dried overnight at 4ºC in the dark. One drop of Vectashield Fluorescence Mounting Medium (Vector Laboratories, Burlingame, CA) and a cover slip were applied to the sections. Slides were stored at 4ºC.

***Epifluorescent Light Microscopy***

ELM samples were excited with a high-pressure mercury vapor lamp and fluorescent signals were filtered with FITC (for Alexa488 signal) and Rhodamine (for Alexa555 and mRFP signals) filters on a Nikon Eclipse E800 (Nikon, Tokyo, Japan) microscope. Images were captured with an attached RT KE color SPOT camera and SPOT software (Diagnostic Instruments, Inc., Sterling Heights, MI). ImageJ (NIH, Bethesda, MD) was used to separate and combine color channels. Adobe Photoshop (Adobe Systems Inc., San Jose, CA) was used to rotate, crop, resize, and adjust contrast, brightness and color levels of images and to assemble ELM figures.
